# A Preliminary Evaluation of the Psychometric Properties of the ARTIC‐10 in Dental Settings: A Cross‐Sectional Study

**DOI:** 10.1002/hsr2.70908

**Published:** 2025-06-11

**Authors:** Freddie O'Donald, Molly Smith, Lindsay‐Jo Sevier‐Guy, Abigail Heffernan

**Affiliations:** ^1^ Department of Special Care Dentistry Dundee Dental Hospital, NHS Tayside Dundee Scotland; ^2^ Department of Clinical Health Psychology NHS Tayside Dundee Scotland; ^3^ School of Health in Social Science University of Edinburgh Edinburgh Scotland; ^4^ School of Dentistry University of Dundee Dundee Scotland

**Keywords:** ARTIC scale, dentistry, psychometric properties, trauma‐informed care

## Abstract

**Background and Aims:**

Trauma‐informed care (TIC) is increasingly recognized in healthcare, including dentistry, for its potential to improve patient outcomes. However, there are currently no validated tools for assessing TIC attitudes among dental professionals. This study aimed to evaluate the psychometric properties of the 10‐item Attitudes Related to Trauma‐Informed Care (ARTIC‐10) scale within a UK dental hospital setting and explore its ability to distinguish between staff with and without prior TIC training.

**Methods:**

A cross‐sectional study was conducted with 43 staff members from a UK dental hospital, including dentists, dental nurses, administrative staff, and technicians. Participants completed the ARTIC‐10 and the Beliefs About Trauma and Trauma‐Informed Care (BAT‐TIC) scale. A subset of participants (*n* = 13) completed the ARTIC‐10 again after 1 month to assess test–retest reliability. Internal consistency, construct validity, test–retest reliability, and group differences were examined.

**Results:**

The ARTIC‐10 demonstrated strong test–retest reliability (intraclass correlation coefficient = 0.891) and moderate construct validity through a significant positive correlation with the BAT‐TIC (*r* = 0.493, *p* = 0.005). TIC‐trained staff scored significantly higher on the ARTIC‐10 (*M* = 5.30, SD = 0.73) than untrained staff (*M* = 4.84, SD = 0.73), *t*(41) = 2.03, *p* = 0.04, with a moderate effect size (*d* = 0.62). However, internal consistency was low (Cronbach's *α* = 0.420), suggesting limitations in scale reliability within this sample.

**Conclusion:**

The ARTIC‐10 shows preliminary promise as a measure of trauma‐informed attitudes among dental staff and may be useful in evaluating training interventions. However, further research with larger, stratified samples is needed to improve internal consistency and assess the scale's broader applicability in dental settings.

## 1 Introduction

The implementation of trauma‐informed care (TIC) in healthcare, including dentistry, is increasingly seen as essential for improving patient outcomes [1]. TIC is a framework for understanding and responding to psychological trauma (a distressing past event), prioritizing safety, trust, collaboration, empowerment, and choice to minimize retraumatization during patient care [2]. It involves recognizing the widespread impact of trauma, identifying signs and symptoms, and integrating trauma awareness into policies, procedures, and interactions with patients. In dental settings, this may include adapting communication styles, reducing sensory triggers, and ensuring patients feel in control during procedures. In dentistry, TIC may reduce distress and enhance patient experiences, particularly for those with trauma histories [3]. However, no standardized measures exist to assess TIC in dental settings, highlighting a gap in understanding its implementation and effectiveness.

The Attitudes Related to Trauma‐Informed Care (ARTIC) scale was originally developed to measure staff attitudes toward trauma‐informed principles across healthcare, education, and social services. The 10‐item short form (ARTIC‐10) has demonstrated good psychometric properties in prior studies with mental health and youth service professionals, and has been used to evaluate trauma‐informed training and organizational readiness for TIC implementation [4]. However, it has not yet been validated for use in dental settings. This study examines the psychometric properties of the ARTIC‐10 among dental staff and evaluates its ability to detect differences in TIC attitudes between trained and untrained staff, providing preliminary evidence for its use as an outcome measure in assessing trauma‐informed care. By exploring both reliability and sensitivity to training‐related differences, the study supports efforts to implement and evaluate trauma‐informed approaches in dentistry.

## 2 Methods

### 2.1 Study Design and Setting

This cross‐sectional study assessed the reliability and validity of the ARTIC‐10 among dental hospital staff. To evaluate test–retest reliability, a subset of participants completed the ARTIC‐10 1 month after the initial survey closed. The study took place in a dental hospital providing both clinical services and professional training.

### 2.2 Participants

All dental hospital staff, including dentists, dental nurses, administrative staff, and technicians, were invited to participate. To ensure representation, recruitment spanned multiple roles and departments. For test–retest reliability, approximately 20% of the total sample was invited to retake the ARTIC‐10 in October 2024, with responses linked using anonymized identifiers. Participation was voluntary, and all responses remained anonymous.

### 2.3 Measures

This study used two measures to evaluate trauma‐informed attitudes and beliefs:

The ARTIC scale is a validated measure designed to assess attitudes toward TIC across five core subscales, including beliefs about behavior, responses to challenging behaviors, and self‐efficacy in TIC implementation. The ARTIC‐10 is the 10‐item version of the scale, developed to measure attitudes related to TIC among staff working in education and health services. Each item is rated on a 7‐point Likert scale, with higher scores indicating more favorable TIC attitudes. The ARTIC‐10 has demonstrated acceptable psychometric properties in healthcare settings but remains unevaluated in dental settings [5].

The *Beliefs About Trauma and Trauma‐Informed Care* (BAT‐TIC) scale was developed by Trauma‐Informed Oregon as a tool to assess awareness and attitudes toward TIC within human service organizations [6]. Although widely used in healthcare, it is primarily a programmatic self‐assessment tool and has not undergone formal psychometric validation. Higher scores indicate a greater awareness of trauma and TIC.

### 2.4 Procedures

Data were collected as part of a service evaluation at Dundee Dental Hospital. Participants completed an anonymous online survey (July–September 2024) covering demographics, prior TIC training, ARTIC‐10, and BAT‐TIC. A randomly selected subtest was invited to retake the ARTIC‐10 in October 2024 to assess test–retest reliability. To examine the scale's sensitivity, participants were grouped based on TIC training attendance.

### 2.5 Data Analysis

Analyses were conducted using SPSS (v25) to assess the ARTIC‐10's psychometric properties:

- *Internal consistency*. Cronbach's alpha (> 0.70) was used to assess reliability.
- *Construct validity*. Pearson's correlation examined the relationship between ARTIC‐10 and BAT‐TIC scores, with positive correlations expected.
- *Test–retest reliability*. Intraclass correlation coefficient (ICC > 0.75) assessed stability over time.
- *Group differences*. Independent samples *t*‐test compared ARTIC‐10 scores between trained and untrained staff to determine if the scale detected differences in TIC attitudes.

All statistical tests were two‐sided, and an alpha level of 0.05 was used to determine statistical significance. Analyses were conducted using IBM SPSS Statistics for Windows, Version 25.0 (IBM Corp., Armonk, NY).

### 2.6 Ethical Considerations

Ethical approval was granted by the NHS Tayside Psychology Department Ethics Review Process (Ref. 2023/PSY/041), with additional approval from NHS Tayside Clinical Governance. Participants provided informed consent, and data were anonymized and securely stored per data protection policies. This study adhered to the Declaration of Helsinki.

## 3 Results

### 3.1 Participant Demographics

A total of 43 participants completed the survey: 20 dentists (46.5%), 12 dental nurses (27.9%), six administrative staff (14%), and five other dental professionals (11.6%), including hygienists and technicians. The average professional experience within the NHS was 10–15 years. Table 1 presents participant breakdowns by training attendance.

**Table 1:** Participant job roles and trauma‐informed care (TIC) attendance.

Job role	Total sample (%)	Attendees (%)	Nonattendees (%)
Dentists	20 (46.5%)	12 (52.2%)	8 (40%)
Dental nurses	12 (27.9%)	7 (30.4%)	5 (25%)
Administration	6 (14%)	2 (8.7%)	4 (20%)
Other (dental hygienists and technicians)	5 (11.6%)	2 (8.7%)	3 (15%)

*Note:* The table presents the number and percentage of participants by job role and TIC training status. “Attendees” refers to participants who self‐reported having completed prior TIC training. Percentages are calculated out of the total sample (*N* = 43) and within each subgroup.

Participants who indicated on the demographic survey that they had previously completed any form of trauma‐informed care training—regardless of format or provider—were categorized as “TIC trained.” No formal training or intervention was delivered as part of the study.

### 3.2 Internal Consistency

Overall, the scale demonstrated low internal consistency (*α* = 0.420). Table 2 presents the Cronbach's alpha values if each item was dropped.

**Table 2:** Cronbach's alpha values for the ARTIC‐10 scale if each item is removed.

ARTIC‐10 item	Cronbach's alpha if item dropped value
1	0.447
2	0.310
3	0.374
4	0.424
5	0.393
6	0.443
7	0.465
8	0.364
9	0.342
10	0.347

*Note:* This table shows how the overall internal consistency (*α*) of the ARTIC‐10 would change if individual items were removed. Lower alpha values indicate reduced reliability, and values above 0.70 are typically considered acceptable for internal consistency.

### 3.3 Construct Validity

Construct validity was assessed via Pearson's correlation between ARTIC‐10 and BAT‐TIC scores. A moderate, significant correlation was found (*r* = 0.493, *p* = 0.005), suggesting that higher ARTIC‐10 scores (more favorable TIC attitudes) corresponded with greater awareness of trauma and TIC.

### 3.4 Test–Retest Reliability

For participants who completed the ARTIC‐10 a month later (*n* = 13), the ICC = 0.891 indicated strong test–retest reliability.

### 3.5 Group Differences

An independent samples *t*‐test compared ARTIC‐10 scores between TIC‐trained (*n* = 23) and TIC‐untrained (*n* = 20) staff. Trained staff scored significantly higher (*M* = 5.30, SD = 0.73) than untrained staff (*M* = 4.84, SD = 0.73), *t*(41) = 2.03, *p* = 0.04, *d* = 0.62, indicating a moderate effect size.

## 4 Discussion

This study provides preliminary evidence on the psychometric properties of the ARTIC‐10 for assessing TIC attitudes among dental staff. Given the lack of validated measures in dental settings, these findings contribute to understanding how TIC is evaluated in this context.

### 4.1 Key Findings

The ARTIC‐10 demonstrated strong test–retest reliability (ICC = 0.891), suggesting it provides stable measurements over time. A moderate, significant correlation with the BAT‐TIC (*r* = 0.493, *p* = 0.005) supports its construct validity, indicating that higher ARTIC‐10 scores align with greater TIC awareness. Additionally, the ARTIC‐10 detected significant group differences, with trained staff scoring higher than untrained staff (*p* = 0.04, *d* = 0.62), suggesting its potential use in evaluating training effectiveness.

However, internal consistency was low (*α* = 0.420), highlighting potential limitations in the scale's reliability within this population. This may reflect context‐specific factors or differences in how TIC is perceived across dental roles.

Although no studies to date have validated the ARTIC‐10 in dental settings, previous research within healthcare environments has shown comparable internal consistency and similar patterns of increased ARTIC scores following training [7, 8]. Our findings appear broadly consistent with these results, suggesting the scale may be sensitive to differences in trauma‐informed attitudes across professional settings. However, as this study involved a small, convenience sample from a single NHS dental service, caution is needed when generalizing findings to dental settings.

### 4.2 Limitations and Future Directions

This study had a small sample size (*n* = 43), limiting generalizability. The inclusion of staff across different roles may also have introduced variability in responses, as these groups differ in their responsibilities and exposure to patient care. Future research should examine the ARTIC‐10 in larger, more diverse dental settings to improve the reliability and validity of findings. Additionally, given the low internal consistency, further analysis is needed to determine whether certain items require modification or an alternative TIC measure may be more appropriate for dental professionals.

As an exploratory study, these findings should be interpreted as hypothesis‐generating rather than confirmatory. No formal power calculation was conducted, and the analyses may have been underpowered to detect some effects, particularly for the test–retest reliability and group comparisons. In addition, the BAT‐TIC used to assess construct validity has not itself undergone formal psychometric validation. Although it was selected due to its relevance to trauma‐informed principles, this limits the strength of conclusions that can be drawn regarding convergent validity and highlights the need for further development of validated tools in this area. Further investigation is needed to establish whether the ARTIC‐10 is a valid tool for measuring TIC implementation in dentistry and to develop standardized frameworks for evaluating TIC in dental care.

### 4.3 Conclusion

This study highlights the need for validated TIC assessment tools in dentistry and provides an initial evaluation of the ARTIC‐10's psychometric properties. Although the findings suggest promise for using the ARTIC‐10 for training evaluation, further research is needed to improve its reliability and establish its broader applicability in dental settings.

## Author Contributions

**Freddie O'Donald:** conceptualization, methodology, formal analysis, project administration, supervision, visualization, writing – review and editing, writing – original draft, investigation, validation. **Molly Smith:** conceptualization, investigation, writing – original draft, writing – review and editing, methodology, visualization, formal analysis, project administration. **Lindsay‐Jo Sevier‐Guy:** conceptualization, writing – review and editing. **Abigail Heffernan:** conceptualization, investigation, writing – original draft, writing – review and editing, methodology, project administration, supervision, resources.

## Disclosure

All authors have read and approved the final version of the manuscript. Dr Freddie O'Donald had full access to all of the data in this study and takes complete responsibility for the integrity of the data and the accuracy of the data analysis.

## Conflicts of Interest

The authors have no conflicts of interest to declare. The authors were provided with a license to use the ARTIC scale but are not affiliated with the team that initially developed the measure.

## 1 Transparency Statement

The lead author Freddie O'Donald affirms that this manuscript is an honest, accurate, and transparent account of the study being reported; that no important aspects of the study have been omitted; and that any discrepancies from the study as planned (and, if relevant, registered) have been explained.

## Data Availability

The data that support the findings of this study are available on request from the corresponding author. The data are not publicly available due to privacy or ethical restrictions.
